# Interface of culture, insecurity and HIV and AIDS: Lessons from displaced communities in Pader District, Northern Uganda

**DOI:** 10.1186/1752-1505-4-18

**Published:** 2010-11-22

**Authors:** Joseph Rujumba, Japheth Kwiringira

**Affiliations:** 1 Department of Paediatrics and Child Health, College of Health Sciences, Makerere University, P.O. Box 7072 Kampala Uganda; 2 Department of Sociology, Kyambogo University, P.O. Box 1, Kyambogo, Kampala, Uganda

## Abstract

**Background:**

Northern Uganda unlike other rural regions has registered high HIV prevalence rates comparable to those of urbanized Kampala and the central region. This could be due to the linkages of culture, insecurity and HIV. We explored community perceptions of HIV and AIDS as a problem and its inter-linkage with culture and insecurity in Pader District.

**Methods:**

A cross sectional qualitative study was conducted in four sub-counties of Pader District, Uganda between May and June 2008. Data for the study were collected through 12 focus group discussions (FGDs) held separately; 2 FGDs with men, 6 FGDs with women, and 4 FGDs with the youth (2 for each sex). In addition we conducted 15 key informant interviews with; 3 health workers, 4 community leaders at village and parish levels, 3 persons living with HIV and 5 district officials. Data were analysed using the content thematic approach. This process involved identification of the study themes and sub-themes following multiple reading of interview and discussion transcripts. Relevant quotations per thematic area were identified and have been used in the presentation of study findings.

**Results:**

The struggles to meet the basic and survival needs by individuals and households overshadowed HIV as a major community problem. Conflict and risky sexual related cultural practices were perceived by communities as major drivers of HIV and AIDS in the district. Insecurity had led to congestion in the camps leading to moral decadence, rape and defilement, prostitution and poverty which increased vulnerability to HIV infection. The cultural drivers of HIV and AIDS were; widow inheritance, polygamy, early marriages, family expectations, silence about sex and alcoholism.

**Conclusions:**

Development partners including civil society organisations, central government, district administration, religious and cultural leaders as well as other stakeholders should mainstream HIV in all community development and livelihood interventions in the post conflict Pader district to curtail the likely escalation of the HIV epidemic. A comprehensive behaviour change communication strategy is urgently needed to address the negative cultural practices. Real progress in the region lies in advocacy and negotiation to realise lasting peace.

## Introduction

HIV and AIDS have had a devastating impact on mankind with varying effects at individual, family, community and institutional levels. Globally, 33 million people were estimated to be living with HIV in 2007[1]. Over the years, HIV concentration remains dominant in Sub-Sahara African countries which accounted for 67% of all people living with HIV in 2007[1]. Uganda has not been spared either, with an estimated HIV prevalence of 6.4% [2]. Results of the Uganda sero-behaviour survey conducted in 2004-05 revealed higher HIV prevalence (8.2%) in the North Central region which includes Gulu, Kitgum, Pader, Lira and Apac districts. This prevalence is comparable to the highest rate of 8.5% in Kampala the capital city and the central region [2]. The high HIV prevalence in the North-Central region could partly be explained by an over 20 year old war between the Government of Uganda and the rebels of the Lord's Resistance Army (LRA)[3]. War and conflict remain major threats to health[4].The Uganda Country progress report on HIV and AIDS 2008[5] highlights co-existence of high HIV prevalence in the conflict areas of Northern Uganda and limited programme coverage as a major challenge. Ill health in conflict areas including the spread of HIV could be exacerbated by negative social cultural practices that increase vulnerability to HIV infection. With relative peace following cessation of hostilities agreement by the Ugandan Government and the rebel group the LRA, several internally displaced persons started returning to their villages[3,5]. This presented need by development agencies including Concern Worldwide in Uganda to develop livelihood programs to address the changing needs of the poor returnees. To ensure that HIV and AIDS issues are well understood and addressed in return villages, we conducted operational research to explore community perceptions on HIV and AIDS as a community problem and its linkages to culture and insecurity in Pader District, Northern Uganda.

## Methods

### Study Area

The study was conducted in the four sub-counties (Pajule, Acholi Bur, Awere and Pader Kilak) in Pader district. These areas were selected purposively being Concern's target areas. Pader district is situated in northern Uganda, bordering the districts of Gulu in the West, Lira in the South, Apac in South west, Abim and Kotido districts in the East and Kitgum in the North. The district is divided into two counties Aruu and Agago, with 17 sub-counties.

Pader district is relatively new. The district was formed in 2001, out of Aruu and Agago counties which were originally part of Kitgum district. The 2002 Uganda national population and housing census estimated the population of the district at 326,338 people[6]. Currently, the population of Pader district is estimated at 481,800 people[7]. Pader district has been devastated by long term insurgency and civil strife for the last 20 years. Government and Non Government Organisation (NGO) interventions exist in the area to reduce human suffering caused by war. One such intervention is that by Concern Worldwide-Uganda which has been implementing humanitarian and emergency projects in water and sanitation, health and hygiene promotion, HIV and AIDS mainstreaming and cash for work in Pader since October 2005. Concern Worldwide is a non-governmental international humanitarian organisation dedicated to the reduction of suffering and working towards the ultimate elimination of extreme poverty in the world's poorest countries. In Uganda, Concern operates in Rakai, Nakasongola, Amuria, Pader districts and Rwenzori and Karamoja regions.

### Study Design

This paper is based on a cross sectional operational research that was conducted in Pader district, northern Uganda between May and June 2008. Operational research has been used extensively in the military, industrial and commercial sectors; and with limited application to health programming[8]. Zachariah et al defines operational research as the search for knowledge on interventions, strategies, or tools that can enhance the quality, effectiveness, or coverage of programmes in which the research is being done[8,9].Moreover, other agencies like the World Health organisation uses the term operational research and implementation research concurrently and includes application of qualitative research methods [10] in addition to the conventional epidemiological study designs. The operational research which is the basis for this paper was conducted as part of the on going Concern Worldwide Uganda activities in Pader district to inform stakeholders on how best to address the livelihood and HIV and AIDS challenge within the post conflict situation. We adopted a qualitative study design for being appropriate in understanding of social processes and concepts from the perspectives of study participants[11,12] informed by their lived experiences[12]. Lived experiences constitute an added value in tailoring programme interventions to suit the local context. This qualitative study was part of the bigger study conducted to assess HIV and AIDS related Knowledge, Attitudes, Practices and Behaviours (KAPB) of the returnees in four Concern target sub-counties of Pader district. Data for the study were collected using 12 focus group discussions (FGDs) held separately; two FGDs for men, six FGDs for women, and four FGDs for the youth (2 for each sex). The sex specific FGDs were intended to allow male and female participants to freely express their concerns about the community priorities in their area and the linkage with regard to culture, HIV and insecurity. The homogeneity of FGDs further aimed at ascertaining the similarities and differences in sub-group perception on the inter-linkages among culture, insecurity and HIV and AIDS. Each focus group had between 6-10 participants. On average, three FGDs were conducted per study sub-county one group for men, women and youth.

Camp leaders identified venues either open space or an office from where we conducted FGDs. Other community members who showed interest in joining the FGDs once the maximum number of 10 had been attained and the discussions had commenced were not allowed to join the FGDs. They were told by the community leader to wait and members of the research team talked to them briefly about the purpose of the study, their concerns and perception on the relationship among culture, insecurity and HIV in their areas. The views from these spontaneous discussions are not included in this paper owing to the large numbers of people in these discussions, the limited time for discussion and difficulties of writing detailed notes. In addition, we conducted 15 key informant interviews with; three health workers, four community leaders at village and parish levels, three persons living with HIV and five district officials. Community leaders particularly camp commandants assisted to mobilise focus group discussion participants and in the identification of key informants at community level while the Pader district health officer and the HIV focal person provided guidance on identification of district level key informants.

We recruited four research assistants who knew both English and Acholi (the main local language spoken in the study district). We trained them for one day and worked with them to pre-test data collection tools. All the four research assistants had prior experience in conducting focus group discussions and interviews. Research assistants worked in pairs one as an FGD facilitator/interviewer and the other as a note taker. They conducted FGDs and interviews in the local language and wrote detailed notes. The two authors of this paper interviewed key informants who could speak English. In addition we facilitated two focus group discussions through a translator. The findings of this process were comparable with those obtained by research assistants. The main issues for discussion were; perception of HIV as a community problem and the influence of insecurity and culture on the spread of HIV. On average, key informant interviews lasted 45 minutes while FGDs took 60-90 minutes. To address the likelihood of inhibition, especially among the youth, FGDs for the female youth were conducted by female researchers and those for male youth were conducted by male researchers. In addition, training of research assistants on techniques of data collection including use of probes helped to make FGDs an effective approach for data collection.

We held daily field review meetings with research assistants to capture emerging issues for follow up and provide guidance for further data collection.

### Data management and analysis

We analyzed data manually using content thematic approach. Following a frame work advanced by Graneheim and Lundman to identify manifest and latent content in the discussion and interview scripts [13]. The two lead investigators read FGD and interview scripts several times independently to identify emerging themes and sub-themes. We then held a joint discussion to compare themes and sub-themes identified; a process that led to development of a unified list of codes for use in data analysis. The major themes identified were; HIV and AIDS were overshadowed by other community needs, insecurity increased the spread of HIV and the social-cultural context increased the risk of contracting HIV. These themes and sub-themes were used to code data from focus group discussions and key informant interviews. Sub-group analysis was done, which involved examining the themes and sub-themes in relation to various categories of FGDs (men, women and youth) and key informants (community and district level KIs) in order to identify similarities and difference in perceptions with regard to culture, insecurity, and HIV and AIDS. We identified verbatim quotations which have been used in presentation of study findings.

### Ethical Issues

We obtained clearance for the study from the Pader District Health Department, sub-county and parish leaders. Verbal consent to participate in the study was obtained from all study participants. Study participants' identifiers were not recorded. The need for confidentiality was emphasized during training of research assistants and conduct of the study. Community members who were found to require guidance on where to seek particular services were guided accordingly. We held a debrief meeting at the end of data collection with members of the district health team and Concern staff to give quick feedback on emerging issues from the field. A study report was written and disseminated to Concern and Pader district stakeholders.

## Results

### Characteristics of study Participants

Study participants included young women (aged 15-30), old women (over 30 years), Men and the youth particularly the unmarried. Most of the community study participants had attained primary education, 1/3 had no education at all and most mentioned subsistence farming as a livelihood. Key informants were mainly district officials, NGO and health facility staff as well as community leaders at village and parish levels.

### HIV and AIDS overshadowed by basic community needs

Throughout the four study sub-counties, community members recognised HIV and AIDS as a community problem. However, in terms of ranking, issues of; water, food, treatment and sanitation (mainly lack of excreta facilities) came before HIV and AIDS. When FGD participants and key informants were asked how HIV and AIDS compared to other community problems and how significant it was in their daily lives, anxiety about satisfying immediate needs of food, shelter, water, medical care and housing were repeatedly evident. Generally, HIV and AIDS were perceived as something for the future since it does not kill immediately compared to hunger. It therefore appeared that the unmet basic needs had over shadowed the fear for HIV and AIDS.

Findings revealed that women were more concerned about meeting the basic needs especially food as illustrated by the voice below.

Yes we know AIDS exists, but we are much more worried about the conditions of our daily lives than HIV. We do not even find enough food to satisfy our stomachs, and what we worry about is what we will eat today (FGD Young women Koyo, Pajule sub-county)

Men were on the other hand more concerned about the wider community needs like water, sanitation facilities and the fear of re-occurrence of insecurity.

HIV is a problem but for now we need water. In the camps where we moved from we had water flowing but in this village people walk long distances to look for water through the bush. Even if you brought a seminar on HIV now people will insist on water as their main problem (Community Leader, Acholi-Bur)

*The main reason why some people have not yet come to the village is lack of water, latrines and fear that insecurity may re-occur but not HIV and AIDS. Those still in camps are better off than us here. Concern is trying to drill a bore hole which will help us a lot when completed (FGD Men Winya, Pader Kilak)*.

These findings show that whereas community members were aware that HIV and AIDS were serious problems, concern to meet the urgent basic needs constituted a greater priority for them. It is quite natural that people cannot think about strategic issues before meeting their practical needs.

Community and district leaders emphasised that more interventions geared towards livelihood improvement were required and once such took place then HIV issues could be mainstreamed there as one of the leaders noted;

What we need to do is to work on community needs like provide water in returnee villages, give people seeds....but when for example a water project is being commissioned then people can be told about HIV and they will listen; or even when they are being given agricultural inputs like seeds (District leader)

What is evident from the above voice is that whereas communities were pre-occupied with pressing basic needs, interventions geared at addressing these needs could also adopt integrated messages and other activities that address HIV as a cross-cutting issue.

### Perceived trends of HIV transmission

Community members and informants believed that HIV and AIDS were on the increase in their communities. This perception was deeply rooted within the complexities and vulnerabilities created by war and the long standing social cultural practices as presented in the subsequent sections of this paper.

### Insecurity and HIV and AIDS

Most study participants were of the view that insecurity had exacerbated the spread of HIV in Pader District. Throughout the discussions the major themes linking HIV to insecurity emerged and these were; congestion in camps associated with moral decadence, rape and defilement, sex for money and other material gains, increasing poverty, and the general breakdown in the health care system including shortage of HIV counselling and testing services.

### Congestion in camps associated with moral decadence

Both FGD participants and key informants reiterated that the war had displaced many people from their villages and pushed them in congested camps where they lived for a long time and feared that their children had been exposed to sexual immorality. To this, one district official noted;

*The war has pushed many people into camps; even children have been born and grown up without proper guidance. You can see the camp environment which exposes children to immorality at an early age due to lack of privacy and some are lured into sex using small gifts (District Official)*.

The linkage between insecurity, social dislocation and sexual immorality was also emphasised by men who observed that;

*Before the war, we used to live in families with our own rules and it was easy to discipline children. It was not easy to have many people mix up like today in camps so the family has lost its value in protecting its members and this has exposed us to HIV (FGD Men)*.

The above findings show that insecurity and displacement in Pader district have greatly weakened the family institutional structure with regard to enforcing discipline among family members especially children thus creating impetus for the risky practices that were perceived to increase the spread of HIV.

Discussions with the youth confirmed the decay of the family structure due to insecurity and its linkage with risky behaviours which exposed the youth to HIV. As one FGD member retorted;

*You know there are many youth who did not go to school or dropped out of school due to the war. They are idle and they can do any thing. Parents no longer have much control over them (FGD male youth)*.

### Poverty, vulnerability and risk of HIV infection

As a result of the war and displacement, most people had lost their property and livelihood bases. This situation forced some people to adopt risky coping strategies in attempts to meet the basic necessities of life. Such strategies included; sex for money and other material gifts, marrying off young girls and marital breakdown in favour of those who had money.

*Desperate men with money hungry for sex in the district engage the services of women and girls who are equally desperate for cash and survival; depending on how much money is available. All these are results of the war and increase the risk of HIV infection (District official)*.

The search for the means to meet the survival needs emerged again and again in discussions with community leaders as a factor that pushed some women into risky sexual practices that increased their vulnerability to HIV infection.

With war people are desperate for survival, so they are struggling for every thing like food and shelter; regardless of the consequences of this exchange which may include HIV (Community Leader, Acholi-Bur)

Men and women in focus group discussions pointed out that poverty which had increased because of the war had resulted into family breakdown and increased promiscuity with potential to increase risk to HIV infection as male and female community members explained.

You know before the war people had their gardens and would cultivate their own food. But we lost all these things. So as women struggle to meet the needs of their families like food, some have been lured into sex by men who have money and it becomes a habit. Such acts increase chances of getting HIV (FGD Men)

Some women, who do not have a strong heart, gave themselves in to the men who had money or those that were in charge of welfare in camps so that they could get some money to buy food for their children. It was not easy; we can blame them but on the other hand they had no choice. If children are crying and they are hungry you can find when you have done what you would not do-if circumstances were different including having sex with men for money (FGD Women)

What emerges from the above voices is that destruction of family livelihoods resulting from conflict had served to push women into a situation characterised by difficult and risky choices which exposed them to risks of HIV infection.

For some men displacement and insecurity meant loss of power and ability to provide for their families. Indeed some men testified that they knew colleagues whose wives had been taken by men who had money and were in influential positions as noted below.

*War is bad. You cannot be a man if you cannot provide for your family. Some men will have to start all over again. Like our friend who had two wives but when we came to the camps he could not provide for them so both wives were taken by other men who had money (FGD Men)*.

*There are also women who do not go away. But they have men aside who give them money. The cause of this is one; the war which made men lose their assets and sources of income. Husbands for such women may not know but they can bring for them HIV (FGD men)*.

### War and sexual abuse-double troubles for women

Study findings also revealed that women and girls were victims of sexual abuse due to war than men. Through focus group discussions and interviews, women and girls were sighted as victims of rape and defilement by rebels and soldiers alike thus increasing the risk of HIV infection. One community leader in Awere observed:

*During the war many women were forced into sex either through rape or in exchange of money and materials*.

Vulnerability to HIV infection was closely knit in role expectations of men, women and children at household level. In order to meet these role expectations different family members adopted varied coping strategies some of which increased the risk of HIV infection. For instance, in order to meet the needs of their families, some women went in for extra marital sex in exchange of food, money, blankets and other household requirements. In this context, failure to meet basic needs resulting from war, poverty and displacement increased vulnerability to HIV infection the level of awareness not withstanding. Men recounted how soldiers, aid workers and business men had preyed on their wives since they had material and financial resources.

There is a man in this village that had 3 wives when we were in the bigger camp but all of them were taken by soldiers because they had money (FGD Men Coo rom)

Some parents were also said to encourage their adolescent daughters to 'be self reliant'; which also included engaging in sex for money as a survival strategy. This was also linked to limited livelihood options in the war torn district.

*There are many young girls in this district who have sex with old men because they want money and other material things for survival (Community leader Pajule)*.

Discussions with the youth also confirmed that family pressures and inability to provide for the needs of the youth was a major push factor into risky sexual relationships.

You can tell your parents you need Vaseline and they tell you that you should find how to buy it yourself since you are a big girl now. What does that mean? So some girls have been pushed into finding men to help them and some men may give them HIV in exchange (FGD female youth)

If you have some money it is not difficult for one to get a girl. You just need to have some little money to buy for her lunch or a dress and she is yours. So the boys and men who have money have many girl friends (FGD male youth)

Role expectations coupled with an insecure environment aggravated exposure to HIV infection particularly for women and girls. For instance, women and adolescent girls had to go and till the fields, collect firewood and water that were inevitable even when they knew that going out to these places was risky, some times involving rape and defilement. Findings from FGDs and key informants confirmed this vulnerability embedded in the struggles by women and girls to meet their family role expectations as noted by one community leader:

We have heard very bad experiences because of the war; you hear a girl or woman was raped on the way to the well to fetch water. Some of those women may end up getting HIV (Community Leader)

### Social-cultural context and HIV and AIDS

When asked as to whether there are any social cultural practices that increased the spread of HIV in Pader district, most of the study participants responded in the affirmative. The main themes that emerged linking HIV spread to the social cultural context include; polygamy, widow inheritance, alcoholism, traditional ceremonies, silence about sex, submissive place of women and resistance to condom use.

Discussions with community members and key informants re-affirmed that widow inheritance, polygamy and early marriages were fuelling the spread of HIV and AIDS in the district. One respondent retorted; *'If I die my brother will represent me'*. Literally meaning that when he dies his brother will inherit his wife. In relation to polygamy one local leader who disclosed having 5 wives defended the practice as '*a gift from God!*'

"Men in this area have many wives and other women a side. A man generally with one woman is seen as if he is not a man enough. HIV is going to finish us" (FGD Women Bolo)

Other risky cultural practices identified were; using un-sterilized instruments by traditional healers and alcoholism which was seen as a norm for most men. The congested camp environment characterised by poverty was again mentioned as a threat to fostering morals in children with regard to sexuality. Abstinence was seen as very hard or even impossible to enforce.

Having many sexual partners, over use of alcohol and early marriages in our setting are major challenges in the fight against HIV and AIDS (District official)

Furthermore, the spread of HIV was linked to silence related to sexuality and HIV status.

*HIV has challenged us, it is not easy to talk about sex in our culture, those are bedroom matters... many adults find it difficult to advise the young ones on HIV prevention (District official)*.

*Even when people test, they prefer their HIV status to remain secret partly because of the secrecy related to sex and the fear of stigma (FGDs men)*.

I would not reveal that I am HIV positive to others, for fear of embarrassment, isolation and discrimination (FGD Female Youth Pajule)

In view of stigma, a person living with HIV observed;

*"Once people know that you are sick, they will soon refuse to associate with you thinking that you are dying any time. By the time you die you will be alone like a stranger and very miserable because we don't have the modern drugs that I hear make people better and can even 'cure' the disease'*.

Cultural functions like marriage, naming of children and rituals related to the birth of twins were also linked to increasing the risk of HIV transmission. Respondents argued that such functions brought many people together and increased avenues for alcohol consumption and casual sex. A common belief that healthy looking persons are likely to be free from HIV re-enforced the risk. It was common to find a woman or man widowed living with HIV getting married over and over again as long as he/she looked healthy. Some traditional healers and herbalists were said to lure their clients particularly women into sexual relationships, a practice with potential to increase the spread of HIV.

It was also strange to note that some people believed that ARVs cure AIDS. To this one elderly man noted that 'AIDS *no longer kills the very rich, they buy modern drugs and recover and begin their lives all over again! It is the poor that are in trouble!'*

On the contrary this notion was dismissed in focus group discussions for the youth and by key informants.

*'HIV and AIDS has no cure; people have no spare parts-you have to test blood, use condoms, be faithful, abstain and be safe! Once you have HIV it will never leave your body until you die (FGD Youth Awere Sub-county)*.

## Discussion

Community members in Pader District highly recognised HIV and AIDS as a community problem. However, the threat of HIV and AIDS was overshadowed by other pressing community needs especially those related to immediate survival such as water, sanitation facilities, food, medical care and shelter. These services were particularly lacking in return villages compared to camps. These findings reflect multiple vulnerabilities which should be central in the design of livelihood enhancement interventions for returnees in Pader district of which HIV and AIDS mainstreaming should be part. It should be noted that lack of social economic rights have been found to augment the risk of HIV infection [14]. The implication here is that HIV and AIDS should be part of all community development, reconstruction and rehabilitation interventions in Pader district which has been largely affected by conflict. Government and civil society actors should go beyond the traditional HIV interventions and address the complex realities obtaining on ground including increasing access to food, income generation and health care services among returnee populations. Opportunities for HIV and AIDS mainstreaming within livelihood projects should be identified and utilised to foster HIV prevention, care and support. For example, community leaders suggested that HIV messages like use of condoms and the need for people to go for HIV counselling and testing could be provided to communities while commissioning community water projects or during the distribution of agricultural inputs. Such insights from communities provide a window of hope to contain the HIV epidemic in the post conflict phase but also challenge development actors to address the multiple vulnerabilities to HIV infection of individuals, households and communities resulting from conflict. Addressing such the community livelihood needs has potential to restore hope for the future among returnees a likely premise for effective behaviour change messages for HIV prevention[15].

The linkage of HIV and insecurity was appreciated by most community members and district officials. To this end, insecurity was understood to have led to increased sexual immorality, displacement of populations, prostitution, rape and defilement, poverty and strain on existing health services which were associated with increased risk of HIV transmission. This community perception is not surprising given the high HIV prevalence in northern Uganda (8.2%) comparable to that of Kampala (8.5%) [2] the capital city of Uganda. These rates remain far higher than those observed in other rural areas of Uganda. This concurs with Fabiani et al and colleagues who noted that, antenatal HIV prevalence in Acholi region of Northern Uganda is higher than the rates reported in other rural areas of Uganda [16]. This is attributed to the social and economic crises resulting from the civil strife that has affected the region since 1986 [14,16]. Conflict has resulted into breakdown of community social structures with negative impact on health indices in the region. For instance the WHO health and mortality survey conducted in Northern Uganda in 2005 [3] revealed that the crude mortality ratio and under five mortality in IDP camps were above emergency thresholds i.e. 1 and 2 per 10,000 per day. The leading causes of mortality were malaria and HIV and AIDS related complications[3]. Our findings re-affirm insecurity as a major threat to health [4]. The recent Uganda Country progress report on HIV and AIDS 2008 [5] highlights co-existence of high HIV prevalence in the conflict areas of Northern Uganda and limited (HIV and AIDS) programme coverage as a major challenge. The vulnerability and need for prioritisation of displaced populations has been highlighted as a precondition in attaining universal access and the Millennium Development Goals[17]. Ciantia in a review paper concluded that conflict creates conducive environment for factors that increase the risk of HIV infection though this may not necessarily translate into higher prevalence [18].Similar arguments have been raised by Becker and colleagues who underscore conflict and society disarray that follow as a unique and conducive environment to increase the spread the HIV epidemic [19]. Indeed IDPs are often isolated and relatively inaccessible characterised by poor government infrastructure and systems[17]. These are directly linked to civil strife[16]. For sustainable impact of development interventions during the post conflict and reconstruction phase, programme designers and implementers should address issues of HIV at community level as part of the development programmes. In addition, the capacity of health care facilities to deliver HIV and AIDS services in the post conflict Pader district should be stepped up.

Study findings revealed that men, women and youth in IDP camps adopted risky practices that increase vulnerability to HIV infection including sex for money and material gain as well as alcoholism because they were desperate owing to the displacement caused by insecurity. Thus broader and long-term community development interventions that target the survival and development needs of formally displaced populations including health, water, sanitation education, food security, income generation and skills building among others are urgently required to restore hope, confidence and self esteem among returnee populations to foster appropriate behaviours and practices for HIV prevention, care and support. Consistent to the proposal of rebuilding individual and community assets and capabilities for HIV prevention in the post conflict Pader district, Barnett and Weston observed that the hope environment can influence HIV and AIDS prevalence. They observe that people with hope for the future and plans for achieving future goals are less likely to engage in activities in the present that put them at risk of illness or death in the future[15].

Contrary to our findings, emerging evidence seem to suggest lower risk of HIV infection among Internally displaced persons (IDPs) when compared to non IDPs [16,19,20]. A likely explanation to this scenario has been offered by Fabiani et al [16] and Westerhaus [14] who argue that, isolation of populations due to insecurity offer a protective effect holding HIV prevalence down despite the high prevalence of rape, transactional sex and lack of preventive interventions [14].Forced displacement has also been associated with reduced social networks in which individuals might be exposed to HIV[20]. Another study carried out in Northern Uganda highlighted issues of overcrowding in camps, over drinking and poverty which affected both physical and mental health of displaced populations in Nothern Uganda[21]. These social determinants work to increase the risk of contracting HIV amongst IDPs. Although it is possible that IDP camps in Pader district, might have had limited population mobility as a protective factor in the spread of HIV; this is gradually fading away as people return to their villages following relative peace in the district. What emerges from this study is that, the risk of HIV infection is likely to increase as people leave camps to their areas of origin if not followed with appropriate HIV prevention and care interventions. In addition, population mobility within and outside the district is also likely to increase, again presenting need to strengthen and expand HIV prevention and care services. The main worry is that reconstruction periods after conflict might even be associated with increased risk of HIV transmission than during conflict [19,20].

Cultural practices like polygamy, widow inheritance, early marriages, alcoholism and silence about sex were linked to the spread of HIV in both IDP camps and return villages. These findings are in agreement with other studies conducted in the Acholi region. For instance, Westerhaus noted that men usually pursue polygamous relationships [14]. The Uganda sero-behaviour survey (2004-05) revealed prevalence of polygamy among married women (33.3%) and men (28.7%) aged 15-49 in northern Uganda which includes Pader district [2]. Polygamy a form of long-term concurrent sexual partnership is one of the key explanations for the high HIV prevalence in Africa [22]. The challenge of concurrent sexual partnerships should be centralised in the development and implementation of HIV and AIDS behaviour change and communication interventions in Pader District. The risk to HIV transmission in northern Uganda is compounded by excessive use of alcohol, a risk factor for unsafe sex [2]. Indeed another study conducted amongst internally displaced persons in Northern Uganda highlighted over drinking as an effect of the war and a cause of ill health[21].

The gender dimensions of vulnerability to HIV infection emerged in this study. Most study participants believed that women and girls were more at risk of HIV infection than men owing to rape, defilement and power imbalances between men and women with regard to sexuality. The gender dimensions of HIV transmission have also been documented by other studies [14,23,24]. The situation is even worsened by the struggles for survival and family sustenance shouldered by women in conflict areas like fetching water and collecting firewood which are likely snares for rape [25]. Livelihood reconstruction programmes should thus address the gender and livelihood based vulnerabilities to HIV infection. For instance, interventions should include components in areas of protection for women and girls from sexual violence, vocational skills training and income generating projects.

Perspectives of community members and district leaders, revealed the perceived inter-linkages among HIV, war and culture which are critical for an effective HIV intervention. Mainstreaming HIV and AIDS into development interventions aimed at addressing the pressing needs of returnees should be a concern for all stakeholders involved in the design and implementation development interventions. However, the qualitative nature of our findings presents a challenge for trends analysis. The findings of this study are in agreement with those of other studies that highlight insecurity and the social cultural factors that increase the vulnerability of populations to HIV infection[14,16,17] and ill health[21]. Community problems might have been exaggerated by some study participants to attract sympathy for support from NGOs. However, triangulation owing to use of multiple sources of data and different data collection methods helped to check the consistency and variations in study findings [26]. Indeed, findings from FGDs with different sub-groups and key informants at community and district levels were generally in agreement on the perceived linkages among HIV, insecurity and culture. The effect of social expectations from NGOs on study findings could be regarded as minimal. Whereas caution should be taken in attempts to use these findings beyond the study communities, the insights generated with regard to HIV and AIDS mainstreaming in post conflict interventions, the need for broader development interventions matched to the varied pressing needs of returnee populations as an entry point for HIV and AIDS and a source of hope for HIV prevention, care and support may be transferable and applicable to the wider Northern Uganda region that has suffered from the same war and its effects. We were not able to interview representatives of special groups including commercial sex workers and victims of rape and gender based violence. Future studies should seek to involve these groups for more understanding of contextual issues that drive vulnerability and risk to HIV infection in post conflict settings.

## Conclusion

Our study has demonstrated that HIV in return villages is currently overshadowed by other pressing community needs like water, food, health care, sanitation and lack of income generation opportunities. The voices of communities and leaders in this study stress the need for HIV mainstreaming in all community development and reconstruction interventions in Pader district and Northern Uganda in general to increase programme relevancy, acceptability and potential for success. Development actors including civil society organisations, central government, district administration, religious and cultural leaders as well as other stakeholders should prioritise HIV mainstreaming in the design, implementation and evaluation of community development interventions in the post conflict reconstruction programmes in Pader district. Addressing the vulnerabilities to HIV infection for women, the youth and men resulting from conflict and displacement should be a central building block in programme design, implementation, monitoring and evaluation. Advocacy and negotiation to end the long standing conflict in northern Uganda should remain a priority in the broader development framework; otherwise interventions will at best continue to fall short of addressing the causes of vulnerability to HIV infection and suffering in the region. Negative cultural practices that increase the risk of HIV infection like; polygamy, widow inheritance, early marriages, alcoholism and silence about sex should be addressed through comprehensive behaviour change and communication interventions. Studies are required to closely monitor changes in incidence and prevalence of HIV in Pader district and northern Uganda in general especially in the post conflict period as more of the formally displaced persons return to their villages. Further operational research to document the effect of HIV mainstreaming in development and livelihood interventions on HIV prevention, care and support is required to improve the effectiveness of development interventions and generate evidence for replication of successful programmes and advocacy for development actors to better meet the needs of conflict affected populations.

## Abbreviations

AIDS: Acquired Immune Deficiency Syndrome; FGDs-Focus group discussions; HIV-Human Immune-deficiency Virus; IDP-Internally Displaced Persons, KAPB-Knowledge, Attitudes, Practices and Behaviours; LRA-Lord's Resistance Army; NGOs: Non Government Organisation.

## Competing interests

The authors declare that they have no competing interests.

## Authors' contributions

Both authors JR and JK participated in study design, data collection, analysis and writing of the manuscript. Both authors reviewed and approved the manuscript for submission.
